# Bone mineral density and bone turnover in adolescent girls with anorexia nervosa: a 3-year retrospective cohort study

**DOI:** 10.3389/fendo.2026.1754392

**Published:** 2026-05-11

**Authors:** Dorota Roztoczyńska, Aleksandra Roztoczyńska, Jerzy Starzyk

**Affiliations:** 1 Department of Pediatric Endocrinology, Chair of Pediatrics, Institute of Pediatrics, Jagiellonian University Medical College, Kraków, Poland; 2 University Hospital in Krakow, Kraków, Poland

**Keywords:** adolescents, anorexia nervosa, bone turnover markers, DXA, osteoporosis

## Abstract

**Background:**

Adolescent girls with anorexia nervosa (AN) are at high risk of reduced bone mineral density (BMD) due to chronic malnutrition, hypogonadism, and endocrine disturbances. Longitudinal studies integrating densitometric, hormonal, and biochemical markers in this population remain limited.

**Objective:**

To retrospectively evaluate bone metabolism in adolescent girls with AN over a three-year period, including assessment of BMD, bone turnover markers, and hormonal and biochemical parameters.

**Methods:**

Thirty-six adolescent girls with AN were followed for up to three years. Lumbar spine BMD (Z-score) was measured annually by DXA. Bone turnover markers (osteocalcin, CTX), hormonal parameters (estradiol, IGF-1, LH, FSH, cortisol, PTH), and calcium metabolism were assessed. Associations with clinical characteristics and treatment status were analyzed.

**Results:**

The greatest BMD loss occurred during the first 12–24 months of illness, with partial recovery at 36 months. Hormonal therapy combined with calcium and vitamin D supplementation was associated with a more favorable BMD trajectory. Higher BMI (used in correlation analyses) was associated with more favorable hormonal profiles (including IGF-1, estradiol and LH), but not with changes in BMD Z-scores. These findings suggest that early skeletal changes may lag behind endocrine recovery during weight restoration. Bone formation markers increased, while bone resorption markers declined. Higher baseline calcium parameters were associated with changes in BMD, potentially reflecting bone resorption.

**Conclusions:**

Bone recovery in adolescent girls with AN depends not only on weight restoration but also on normalization of endocrine function. The effect of nutritional rehabilitation on bone appears to be mediated through hormonal recovery, while skeletal improvement may lag behind. Monitoring bone turnover markers and calcium homeostasis may provide additional clinical insight.

## 1 Introduction

Anorexia nervosa (AN) is a severe eating disorder characterized by deliberate restriction of food intake, leading to significant weight loss. Affected individuals experience intense fear of gaining weight and a distorted perception of body shape and weight, which reinforces pathological eating behaviors (1). The disorder predominantly affects females, typically emerging during adolescence or early adulthood (2). Recent studies have shown that individuals with anorexia nervosa frequently experience elevated levels of school and family stress, higher rates of psychoactive substance use, and a greater incidence of self-destructive behaviors, underscoring the complex psychological burden associated with the disorder (3). In recent decades, the number of AN diagnoses has increased, particularly among adolescents and young females, likely due to earlier detection and revisions in diagnostic criteria, such as those introduced in the DSM-5-TR (4).

Global meta-analyses estimate the prevalence of AN in children and adolescents at approximately 0.6 % (5). The condition is associated with high mortality rates (SMR ≈ 5.3), resulting from chronic malnutrition as well as medical and psychiatric complications (6).

One of the most serious long-term consequences of AN is the development of osteoporosis (7). In pediatric populations, osteoporosis is assessed not only through bone mineral density (BMD) measurements but also by evaluating bone development dynamics and bone age. Contemporary definitions of pediatric osteoporosis (ISCD) require either the presence of a vertebral compression fracture (not related to high-energy trauma or malignancy), independent of BMD, or a reduced BMD/BMAD (bone mineral apparent density, BMAD) Z-score (≤ –2.0 SD) accompanied by a clinically significant fracture history, rather than relying solely on densitometry (8). In adolescents with secondary causes of bone loss, such as AN, a Z-score ≤ –2.0 may indicate an increased risk of osteoporosis and fractures.

Assessment of bone metabolism extends beyond densitometry. Biochemical bone turnover markers (BTMs) provide insights into the balance between bone formation and resorption, indirectly reflecting osteoblast and osteoclast activity (9). Classic formation markers include osteocalcin (OC), N-terminal propeptide of type I procollagen (P1NP), and bone-specific alkaline phosphatase (ALP), while resorption markers include C- and N-terminal telopeptides of type I collagen (CTX, NTX) and pyridinolines (PYD, DPD) (10).

Emerging markers, such as osteoprotegerin (OPG), RANKL (Receptor Activator of Nuclear Factor κB Ligand), sclerostin, and Dkk-1 (Dickkopf-related protein 1), offer additional insights into the regulatory mechanisms controlling bone formation and resorption (11, 12). While OC and CTX remain foundational BTMs for monitoring early changes in bone metabolism, these markers continue to provide valuable clinical and research information regarding bone health in adolescent girls with AN (13).

In patients with AN, chronic energy deficiency, malnutrition, and secondary hypogonadism inhibit osteoblast activity while maintaining or enhancing bone resorption (14). Evaluating BTMs therefore serves not only as a diagnostic tool but also as a prognostic indicator, helping to monitor osteoporosis risk and treatment efficacy. Disturbances in bone metabolism remain a major clinical issue in adolescents with AN (7, 14).

The aim of the study was a retrospective, comprehensive evaluation of bone metabolism in adolescent girls with anorexia nervosa, including assessment of bone mineral density, biochemical and hormonal parameters, and bone turnover markers over a three-year period of treatment.

## 2 Materials and methods

### 2.1 Study cohort

This retrospective study included 36 adolescent girls diagnosed with AN. The participants’ age ranged from 11 years 10 months to 20 years 3 months (mean 15.3 years), and the duration of illness ranged from 2 to 42 months (mean 12.0 months). At baseline, one girl had ongoing menstruation, eight had not yet experienced menarche, and 27 had secondary amenorrhea lasting 3–36 months (mean 11.1 months). BMI-for-age Z-scores (zBMI) ranged from −3.59 to +1.08 (mean −1.56), according to WHO growth standards. The follow-up period ranged from 12 to 36 months (mean 34.7 months), with 33 girls completing the three-year observation. Three participants did not complete the follow-up, as they discontinued clinic visits after achieving weight normalization (**Table 1**).

**Table 1:** Baseline characteristics of the AN and control groups.

Variable	AN group (N = 36)	Control group (N = 37)
Age (years), mean ± SD	15.3 ± 2.0	15.3 ± 1.5
Age, median (min–max)	16.1 (11.83–20.25)	15.1 (12–18.91)
BMI-for-age Z-score (zBMI), mean ± SD	−1.56 ± 1.01	0.58 ± 0.52
BMI-for-age Z-score (zBMI), median (min–max)	−1.55 (−3.59 – +1.08)	0.57 (−0.63 – +1.87)
Disease duration (months), mean ± SD	12.0 ± 9.1	–
Disease duration, median (min–max)	10 (2–42)	–
Amenorrhea / time since menarche (months), mean ± SD	11.1 ± 7.1	28.1 ± 13.3
Amenorrhea / time since menarche, median (min–max)	9 (3–36)	25.5 (3–58)

BMI-for-age Z-scores (zBMI) were calculated according to WHO growth standards.

### 2.2 Inclusion criteria

Diagnosis of AN was established according to DSM-IV-TR criteria. All patients were evaluated and monitored by a pediatric psychiatrist, who confirmed the diagnosis and determined the appropriate course of treatment.

From an initial cohort of over 50 patients, only those meeting strict inclusion criteria were enrolled. Patients were excluded if they had other somatic disorders affecting bone mineralization, participated in competitive sports, performed regular strenuous exercise, received systemic or inhaled corticosteroids, had lactose or milk protein intolerance, had other chronic diseases, or had confirmed purging behaviors (self-induced vomiting or laxative use). All included patients were exempt from physical education and were advised to refrain from exercise until normalization of body weight. Additionally, patients who did not provide informed consent were excluded. Detailed medical history included dietary intake, physical activity, pubertal development, comorbidities, medications, and fracture history.

### 2.3 Therapeutic interventions

All patients received structured dietary management and family-based systemic psychotherapy, including both individual and family therapy. Nutritional rehabilitation consisted of gradual caloric increase, with careful monitoring of dietary intake and electrolyte levels to prevent refeeding syndrome. Additionally, 11 patients received clomipramine, which, according to current evidence, does not affect bone mineral density. Hormonal and pharmacological interventions for bone health were administered as clinically indicated. Among participants, 9 received only calcium and vitamin D_3_ supplementation. Eleven patients received additional hormone replacement therapy lasting from 3 to 24 months (mean 9.34 months), and 16 received no pharmacological treatment due to normal baseline BMD or lack of adherence.

### 2.4 Subgroup analysis was performed according to two criteria:

#### 2.4.1 Baseline DXA Z-scores

Ia: normal (> –1 SD, N = 16).

Ib: moderately reduced (–1 to –2 SD, N = 8).

Ic: severely reduced (< –2 SD, N = 12).

#### 2.4.2 Treatment status

IIa: no pharmacological therapy (N = 16).

IIb: treatment with calcium and vitamin D_3_, with or without hormonal therapy (N = 20).

### 2.5 Clinical and anthropometric assessments

Physical examinations were performed daily during hospitalization and monthly after discharge as part of routine clinical care. For the purpose of this study, comprehensive anthropometric assessments, including evaluation of growth and BMI, were conducted every 6 months.

BMI-for-age Z-score (zBMI) was calculated according to WHO growth standards and used to describe nutritional status at baseline and at the end of follow-up. Due to the retrospective nature of the study and the need for individual reference data at each time point, zBMI could not be consistently calculated for all intermediate measurements. Therefore, both zBMI (where available) and absolute BMI values were used in the analyses, with correlation analyses based on absolute BMI to preserve sample size and ensure comparability across time points.

Pubertal stage was assessed using the Tanner scale, and skeletal age was determined annually from a left-hand X-ray.

### 2.6 Bone densitometry

Lumbar spine BMD (L2–L4, AP) was measured annually using dual-energy X-ray absorptiometry (DXA) with a Lunar DPX-IQ densitometer. BMAD was calculated according to Kroger’s formula, and Z-scores were calculated using the manufacturer-provided age- and sex-specific reference data. Additionally, Z-scores adjusted for bone age were assessed. Bone mineral content (BMC) was also derived from DXA measurements but was not included in the primary analysis, as absolute measures are strongly influenced by body size and may be less informative in adolescents with anorexia nervosa. BMAD was calculated to account for bone size; however, Z-scores were used as the primary outcome parameter for age- and sex-adjusted assessment of bone status.

Changes in Z-score were analyzed as the primary outcome parameter, as this measure reflects bone mineral status relative to normative values while accounting for skeletal maturation.

The coefficient of variation (CV%) for lumbar spine DXA measurements is typically reported to be in the range of 1–2%, although formal precision assessment was not performed in this retrospective study. Measurement reliability was ensured through routine quality-control procedures, including regular phantom calibration and internal monitoring of measurement stability throughout the study period. All measurements were performed in a single center by the same experienced operator.

### 2.7 Hormonal assessments

Blood samples were collected every 6 months at 8:00 a.m. after an overnight fast. Serum estradiol, LH, FSH, PTH, IGF-1, and cortisol were measured using radioimmunoassay (RIA; Estradiol [125I] Spectria, PTH-IRMA Biosource, SM-C-RIA-CT Biosource, Cortisol [125I] Spectria) or chemiluminescence (LH, FSH; ASC 180 plus, Bayer), as appropriate. Urinary free cortisol was measured by RIA (Cortisol [125I] Spectria) after extraction.

### 2.8 Biochemical and bone turnover assessments

Biochemical parameters, including total calcium and alkaline phosphatase, were determined by dry chemistry on a Vitros 700 analyzer (Johnson & Johnson). Bone turnover markers (BTMs)—N-MID osteocalcin (OC) and serum CrossLaps (CTX)—were assessed using one-step ELISA kits (Osteometer BioTech). Twenty-four-hour urine collections were analyzed for calcium, phosphate, magnesium, and creatinine excretion by dry chemistry (Vitros 700), and the calcium-to-creatinine ratio was calculated.

### 2.9 Follow-up schedule

All hormonal, biochemical, and bone turnover assessments were repeated at 6, 12, 18, 24, 30, and 36 months.

### 2.10 Statistical analysis

Data were analyzed using Statistica 6.0 PL and SPSS. Normality was assessed using the Kolmogorov–Smirnov test. Because data were not normally distributed, nonparametric tests were applied. Descriptive statistics included medians and ranges. Between-group comparisons were performed using Mann–Whitney and Kruskal–Wallis tests. Spearman correlation was used to assess associations between variables. A p-value < 0.05 was considered statistically significant. Because of the exploratory nature of the study and the limited sample size, no formal correction for multiple comparisons was applied, and results should be interpreted cautiously.

### 2.11 Control group

Since Polish reference ranges for BTMs in adolescents are lacking, a control group of 37 girls of similar age with normal weight and regular menstruation (except three premenarchal) was included. Baseline marker levels were compared with the AN cohort (**Table 1**).

### 2.12 Ethics statement

The study was conducted retrospectively and approved by the Bioethics Committee of the Jagiellonian University (KBET/149/B/2000) in accordance with the Declaration of Helsinki.

## 3 Results

### 3.1 BMI at baseline and during 3-year follow-up

At baseline, the mean age of the 36 girls was 15.3 years. The mean BMI-for-age Z-score (zBMI) was −1.56, ranging from −3.59 to +1.08.

**Figure 1:**
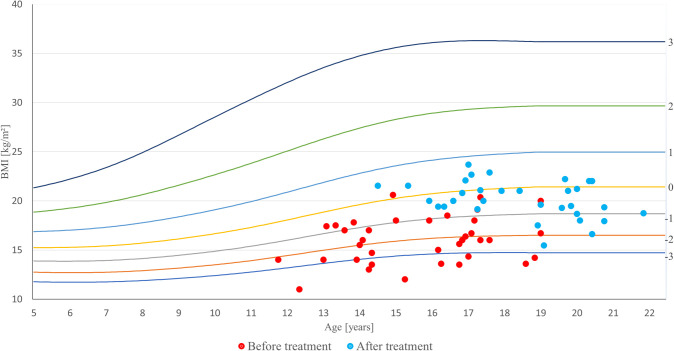
BMI-for-age Z-score (zBMI) in adolescent girls with anorexia nervosa before and after treatment.

During the three-year follow-up, zBMI increased significantly (p < 0.01) (**Figure 1**). At the end of the follow-up, among the 33 girls who completed the observation, the median zBMI was −0.7, ranging from −1.92 to +1.3.

Despite improvement in zBMI, no direct association was observed between BMI (used in correlation analyses) and lumbar spine BMD Z-scores (p > 0.05).

### 3.2 Bone mineral density (Z-score) at baseline and during 3-year follow-up

**Figure 2:**
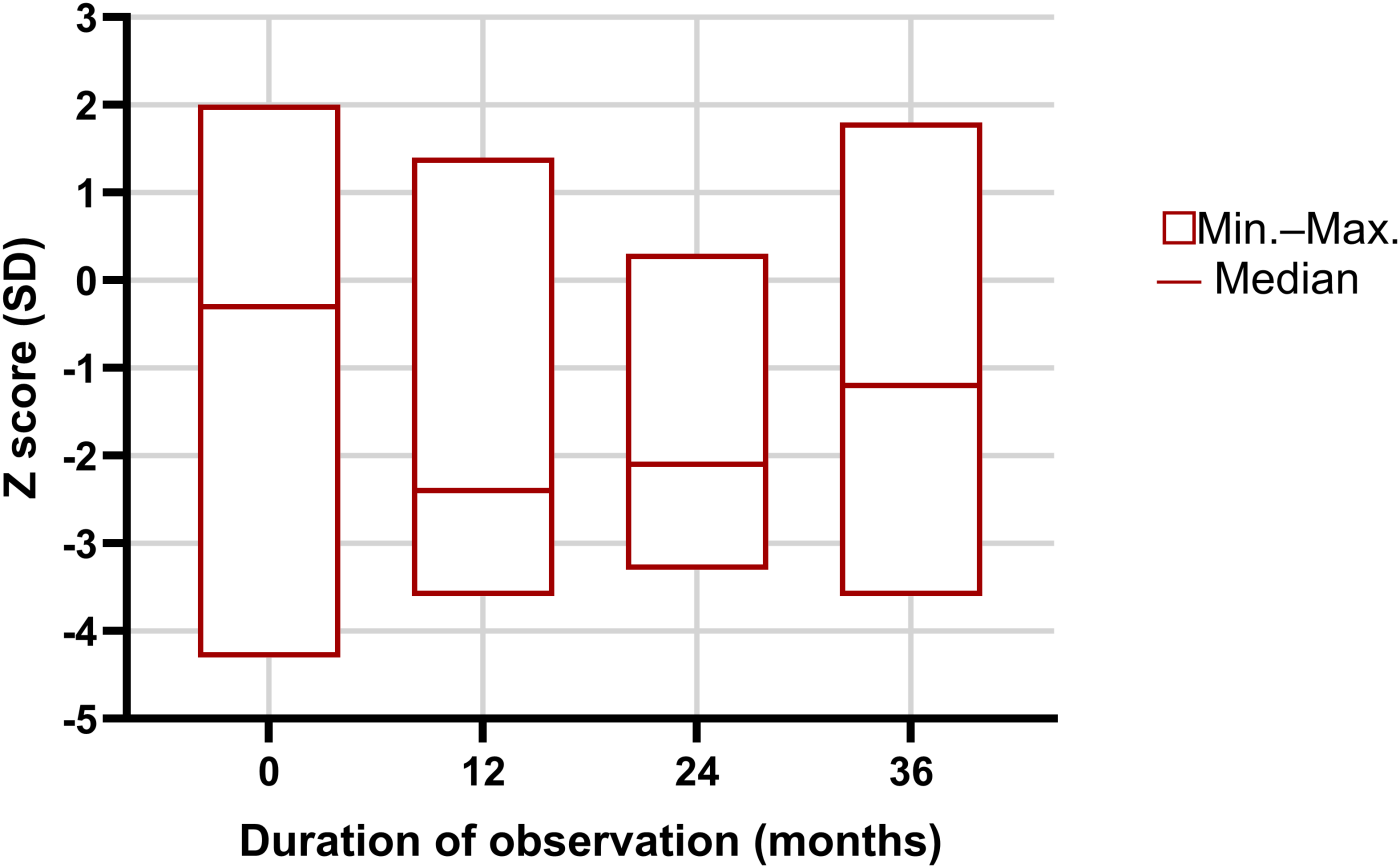
Longitudinal changes in lumbar spine Z-scores in adolescent girls with anorexia nervosa over the three-year follow-up. Significant differences were observed between time points (p < 0.05 for 0 vs 12 months, 12 vs 36 months, and 24 vs 36 months).

The initial lumbar spine densitometry in the study group revealed reduced bone mineral density, with a median Z-score of –1.2 SD. After one year of observation and treatment, a significant decline in Z-score was observed (–2.4 SD, p < 0.05). At 24 months, the median Z-score was –2.1 SD, while at 36 months, in 33 patients who completed follow-up, the median Z-score improved to –1.2 SD, showing a significant increase compared to the 12- and 24-month measurements (p < 0.05) (**Table 2**, **Figure 2**).

**Table 2:** Changes in lumbar spine Z-score over time in the overall AN cohort and according to baseline bone density subgroups (Ia: normal, > −1 SD, N = 16; Ib: moderately reduced, −1 to −2 SD, N = 8; Ic: severely reduced, < −2 SD, N = 12).

Time (months)	Overall Z-score, median (range)	Ia: normal	Ib: moderately reduced	Ic: severely reduced
0	-1.2 (-4.3 – 2.0)	0.50 (-1 – 2.0)	-1.40 (-1.7 – -1.1)	-2.65 (-4.3 – -2.2)
12	-2.4 (-3.6 – 1.4)	-0.25 (-2.7 – 1.4)	-2.20 (-2.5 – -0.5)	-2.90 (-3.6 – -2.5)
24	-2.1 (-3.4 – 0.3)	-0.90 (-2.7 – 0.3)	-2.05 (-2.8 – -1.9)	-2.60 (-3.4 – -1.2)
36	-1.2 (-3.6 – 1.8)	-0.20 (-2.2 – 1.8)	-1.70 (-2.6 – -0.1)	-2.20 (-3.6 – -0.9)
Δ Z-score	–	Ia: –0.48 (–2.32 – 0.50)	Ib: –0.30 (–0.9 – 1.4)	Ic: 0.60 (–0.6 – 1.8)

Δ Z-score indicates change from baseline to 36 months. Differences between subgroups were statistically significant (Kruskal–Wallis test, p = 0.0031; Ia vs Ic, p = 0.001). Subgroup sizes were small; results should be interpreted as descriptive.

### 3.3 Subgroups Ia–Ic (baseline Z-score and longitudinal changes)

Across all subgroups, the lowest Z-scores were observed after 1 and 2 years of follow-up. In subgroup Ia, Z-scores declined during the first two years and increased slightly after three years, but did not reach baseline levels. In subgroup Ib, a similar pattern was observed: Z-scores decreased initially and only showed partial recovery by year three. In subgroup Ic, Z-scores improved steadily over the 3 years, although the median value at the end of observation still indicated reduced bone mass (**Table 2**).

Overall, in girls with initially reduced bone mass (Ib and Ic), the decline in Z-score occurred primarily after the first year, followed by gradual improvement. In contrast, girls with initially normal bone mass (Ia) experienced a decrease in Z-score during the first two years, with modest recovery observed only after three years. Statistically significant differences between Ia and Ic (p = 0.001) should be interpreted cautiously due to the small sample sizes in these subgroups. (**Table 2**).

### 3.4 Treatment subgroups IIa vs IIb (baseline and longitudinal DXA Z-score results)

Comparing subgroups according to treatment, girls in group IIa (no calcium, vitamin D_3_, or cyclic estrogen–progesterone therapy, N = 16) showed a decline in Z-score over the 3-year follow-up, whereas a more stable or improving trajectory was observed in group IIb (pharmacological treatment, N = 20).

Importantly, the groups differed substantially at baseline, with group IIb having markedly lower initial Z-scores and longer duration of secondary amenorrhea, reflecting more severe disease.

**Figure 3:**
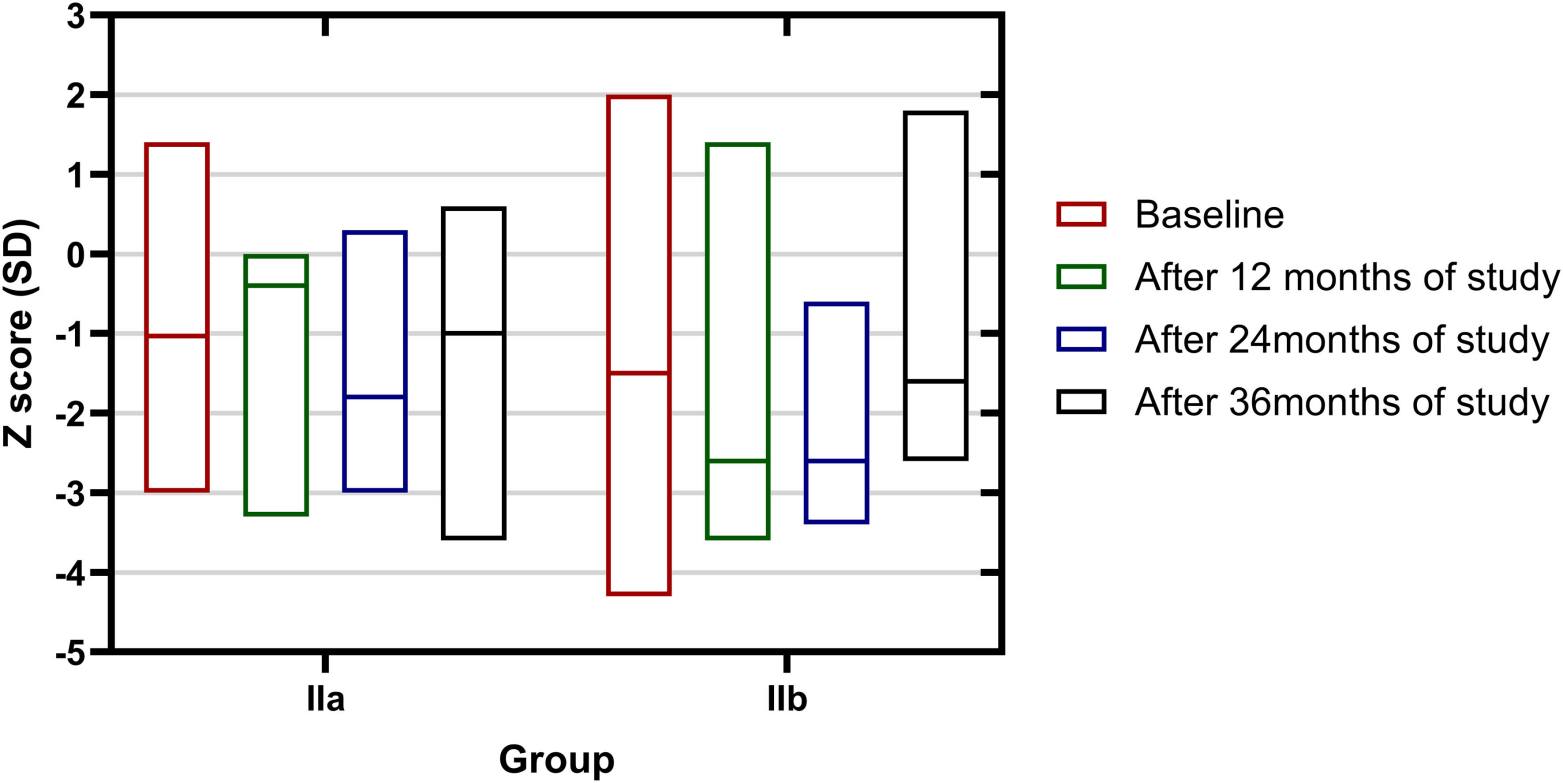
Changes in lumbar spine Z-score in treatment subgroups IIa and IIb over the three-year follow-up.

Changes in Z-score over time differed between groups (**Table 3**, **Figure 3**); however, these findings should be interpreted with caution due to small subgroup sizes, lack of randomization, and significant baseline imbalance.

**Table 3:** Lumbar spine Z-score over 36 months according to treatment status (IIa: no pharmacological treatment, N = 16; IIb: pharmacological treatment, N = 20).

Time (months)	IIa (no treatment, N=16), median (range)	IIb (treatment, N=20), median (range)
0	-0.5 (-3.0 – 1.4)	-2.2 (-4.3 – 2.0)
12	-0.4 (-3.3 – 0.0)	-2.6 (-3.6 – 1.4)
24	-1.9 (-3.0 – 0.3)	-2.6 (-3.4 – -0.6)
36	-0.9 (-3.6 – 0.57)	-1.7 (-2.6 – 1.8)
Δ Z-score	–0.50 (–2.32 – 0.50)	0.50 (–1.8 – 1.8)

Δ Z-score indicates change from baseline to 36 months. Differences between groups were statistically significant (Mann–Whitney U test, p < 0.01). The groups differed at baseline in Z-score and disease severity; therefore, comparisons should be interpreted as descriptive and do not imply causal treatment effects.

In group IIa, the median Z-score was –0.5 SD at baseline, declined during the first two years, and showed partial recovery after three years (median –0.9 SD at 36 months). In contrast, group IIb showed a modest increase in Z-score over the same period (median change +0.50 SD).

Given that treatment allocation was based on clinical indications, the observed differences likely reflect a combination of underlying disease severity, regression to the mean, and potential treatment effects. Therefore, these comparisons are descriptive and do not allow causal inference regarding the efficacy of hormonal therapy.

### 3.5 Correlations at baseline

**Figure 4:**
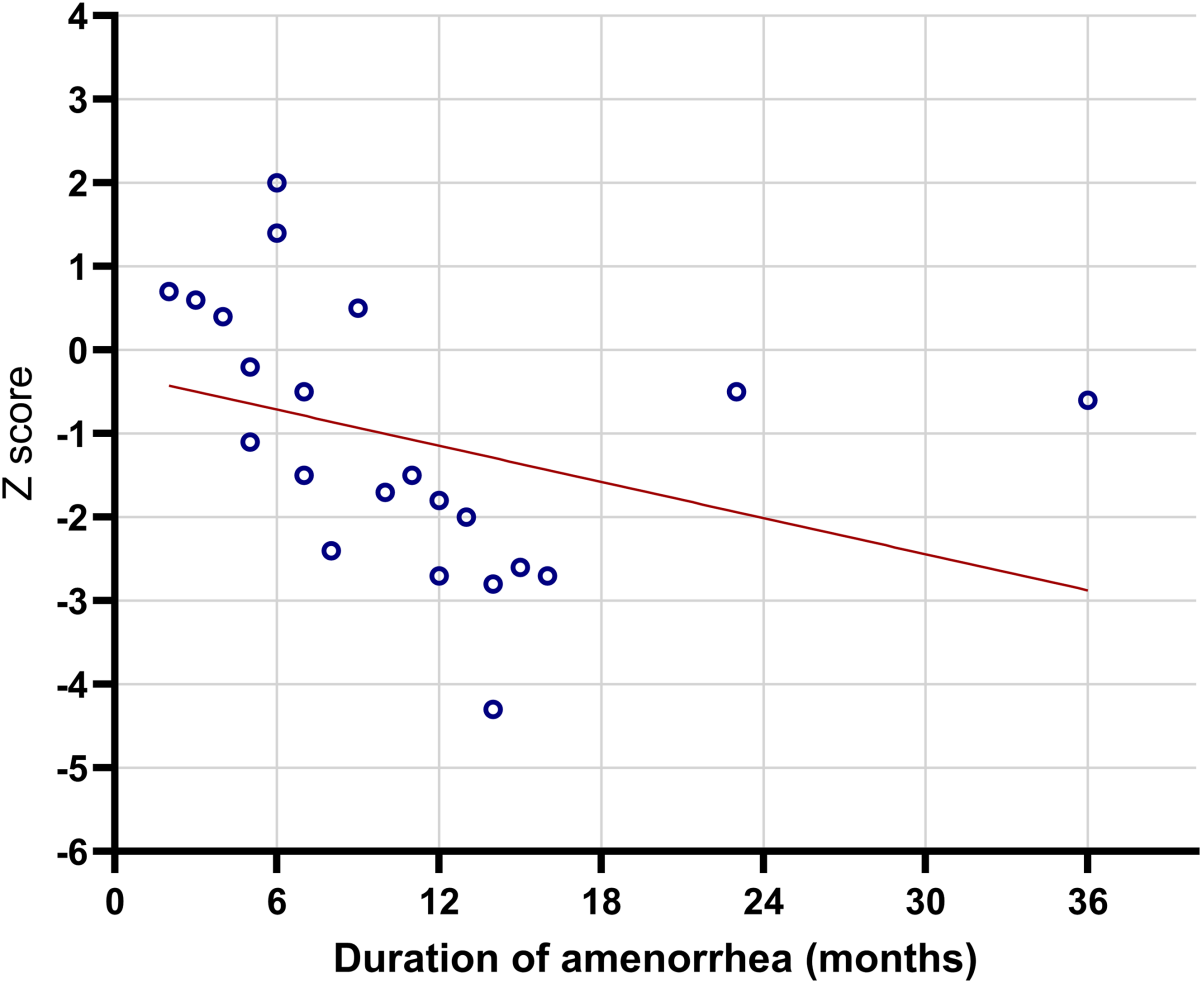
Relationship between lumbar spine Z-score and the duration of secondary amenorrhea in girls with anorexia nervosa at the start of the study (p = 0.01).

At study initiation, Z-score was negatively correlated with the duration of secondary amenorrhea, indicating that longer amenorrhea was associated with lower bone mass (**Table 4**, **Figure 4**). No significant association was observed between Z-score and BMI (used in correlation analyses).

**Table 4:** Correlations between clinical, hormonal, and biochemical parameters at baseline.

Category	Variables	N	r	p
Hormonal and metabolic parameters	**Estradiol & BMI**	**36**	**0.634**	**< 0.001**
	**Estradiol & FSH**	**36**	**0.533**	**0.001**
	**Estradiol & LH**	**36**	**0.359**	**0.034**
	**IGF-1 & BMI**	**36**	**0.542**	**0.001**
	**IGF-1 & FSH**	**36**	**0.435**	**0.010**
	**IGF-1 & LH**	**36**	**0.571**	**< 0.001**
	**24-h urinary cortisol & IGF-1**	**36**	**−0.614**	**< 0.001**
	**24-h urinary cortisol & FSH**	**36**	**−0.475**	**0.009**
	**Serum CrossLaps (CTX) & serum cortisol**	**32**	**0.367**	**0.042**
	**Serum CrossLaps (CTX) & 24-h urinary cortisol**	**32**	**−0.527**	**0.005**
Bone turnover markers	**Serum CrossLaps (CTX) & IGF-1**	**32**	**−0.351**	**0.049**
	**Serum CrossLaps (CTX) & LH**	**32**	**−0.456**	**0.009**
	**Serum CrossLaps (CTX) & FSH**	**32**	**−0.474**	**0.006**
	**N-Mid osteocalcin & IGF-1**	**32**	**0.378**	**< 0.001**
	**N-Mid osteocalcin & estradiol**	**32**	**0.194**	**0.027**
	**N-Mid osteocalcin & ALP**	**32**	**0.367**	**< 0.001**
	**N-Mid osteocalcin & urinary cortisol**	**32**	**−0.295**	**0.002**
	**N-Mid osteocalcin & urinary calcium-to-creatinine ratio**	**32**	**−0.212**	**0.022**
Calcium and phosphate metabolism	**ALP & serum calcium**	**36**	**0.494**	**0.003**
	**ALP & urinary calcium**	**34**	**−0.505**	**0.010**
	**ALP & urinary calcium-to-creatinine ratio**	**34**	**−0.672**	**< 0.001**
Clinical parameters	**Duration of secondary amenorrhea & urinary calcium**	**27**	**0.439**	**0.046**
	**Duration of secondary amenorrhea & Z-score**	**27**	**−0.473**	**0.012**

Spearman correlation coefficients are presented. Multiple comparisons were not adjusted; results should be interpreted as exploratory.

Both total serum calcium and urinary calcium-to-creatinine ratio were significantly associated with Z-score: higher serum calcium was linked to lower Z-score, while higher urinary calcium excretion correlated positively. These findings likely reflect increased bone resorption rather than primary disturbances in calcium metabolism.

Anabolic hormones, such as estradiol and IGF-1, were positively associated with BMI. IGF-1 and LH also showed positive correlations. In contrast, 24-hour urinary cortisol was negatively correlated with IGF-1 and FSH.

At baseline, when body weight deficit was greatest, BTMs were markedly altered compared with healthy controls. Serum CrossLaps, a marker of bone resorption, was elevated, whereas N-Mid Osteocalcin, a marker of bone formation, was markedly reduced. Alkaline phosphatase values were at the lower end of the age-specific reference range. Median N-Mid Osteocalcin in girls with anorexia nervosa was significantly lower than in healthy controls, indicating severely suppressed bone formation at the time of maximal weight deficit (**Table 5**).

**Table 5:** Correlations between clinical, hormonal, and biochemical parameters during the 3-year follow-up.

Variables	N	r	p
Z-score & serum calcium	36	−0.504	0.002
Δ Z-score & serum calcium	33	0.366	0.035
Δ Z-score & urinary calcium-to-creatinine ratio	32	0.374	0.034
IGF-1 & BMI	36	0.401	< 0.001
Estradiol & BMI	36	0.246	0.001
IGF-1 & N-Mid osteocalcin	32	0.378	< 0.001
IGF-1 & CrossLaps (CTX)	32	−0.351	0.049
Estradiol & N-Mid osteocalcin	32	0.194	0.027
ALP & serum calcium	36	0.494	0.003
ALP & urinary calcium-to-creatinine ratio	34	−0.672	< 0.001
Serum calcium & 24-h urinary cortisol	36	−0.195	0.021
Urinary calcium-to-creatinine ratio & 24-h urinary cortisol	32	0.208	0.017
CrossLaps (CTX) & serum cortisol	32	0.367	0.042
CrossLaps (CTX) & 24-h urinary cortisol	32	−0.527	0.005
CrossLaps (CTX) & LH	32	−0.456	0.009
CrossLaps (CTX) & FSH	32	−0.474	0.006
24-h urinary cortisol & IGF-1	36	−0.614	< 0.001
24-h urinary cortisol & FSH	36	−0.475	0.009
Duration of secondary amenorrhea & Z-score	27	−0.473	0.012
Duration of secondary amenorrhea & urinary calcium	27	0.439	0.046

Spearman correlation coefficients are presented. Due to repeated measurements, results should be interpreted as exploratory. Multiple comparisons were not adjusted.

CrossLaps correlated positively with serum cortisol and negatively with urinary cortisol, whereas N-Mid Osteocalcin showed positive correlations with IGF-1, estradiol, and ALKP, reflecting the interaction between hormonal status and bone turnover.

These findings may reflect the adaptive role of hypercortisolemia in response to chronic energy deficiency, which contributes to maintenance of metabolic homeostasis but has unfavorable effects on bone metabolism.

**Figure 5:**
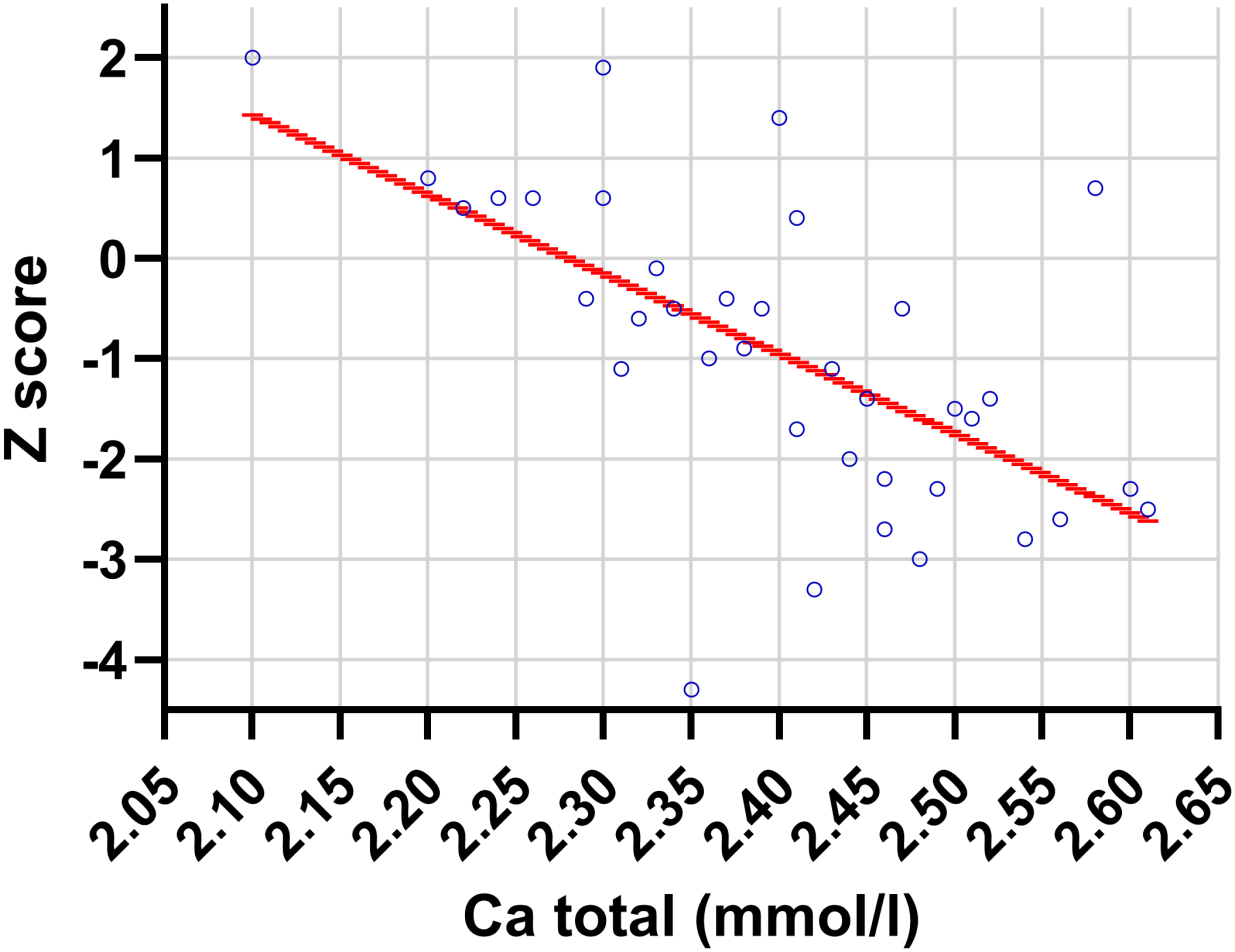
Relationship between lumbar spine Z-score and total serum calcium concentration in adolescent girls with anorexia nervosa at baseline (p = 0.001).

At baseline, total serum calcium was significantly negatively correlated with Z-score in girls with anorexia nervosa (r = −0.504, p = 0.002), indicating that higher serum calcium levels were associated with more severe bone loss (**Figure 5**).

### 3.6 Longitudinal changes and correlations over 3 years

Higher BMI was consistently associated with increased concentrations of estradiol (r = 0.246, p = 0.001), IGF-1 (r = 0.401, p < 0.001), and LH (r = 0.267, p < 0.001), indicating gradual recovery of the gonadal and somatotropic axes. At the same time, BMI was inversely correlated with serum cortisol at 8:00 a.m. (r = −0.320, p < 0.001) and 24-hour urinary cortisol (r = −0.215, p = 0.01), reflecting a progressive normalization of stress-axis activity. Notably, among the 20 girls who received cyclic estrogen-progesterone therapy, estradiol levels rose as expected; however, statistical associations between BMI and estradiol remained significant even after accounting for hormonal therapy. Although BMI SDS increased significantly during follow-up, no significant association was observed between BMI (used in correlation analyses) and lumbar spine BMD Z-scores, indicating that weight restoration alone did not directly translate into recovery of age-adjusted bone mineral density.

IGF-1 remained strongly associated with BMI, LH, alkaline phosphatase, and N-Mid Osteocalcin throughout the observation period (r = 0.378, p < 0.001), highlighting the restoration of anabolic bone processes during nutritional rehabilitation. Serum and urinary cortisol showed negative correlations with IGF-1, LH, and bone formation markers, and positive associations with the bone resorption marker CrossLaps (r = 0.367, p = 0.042 for serum; r = −0.527, p = 0.005 for urinary), confirming the inhibitory and catabolic influence of cortisol on bone metabolism.

Importantly, changes in lumbar spine Z-score over the three years were positively associated with both serum calcium concentrations (ΔZ-score vs serum Ca: r = 0.366, p = 0.035) and urinary calcium excretion expressed as Ca/creatinine ratio (ΔZ-score vs Ca/creatinine: r = 0.374, p = 0.034). Baseline total serum calcium was negatively correlated with Z-score (r = −0.504, p = 0.002), indicating that higher serum calcium levels were observed in patients with more severe bone loss. These findings suggest that alterations in calcium homeostasis—reflected by both serum and renal calcium handling—may mirror the degree of bone resorption and carry prognostic value for subsequent skeletal recovery. Additionally, urinary cortisol was negatively correlated with serum calcium (r = −0.195, p = 0.021) and positively correlated with urinary calcium excretion expressed as the calcium-to-creatinine ratio (r = 0.208, p = 0.017) (**Table 6**).

**Table 6:** Bone turnover markers in adolescent girls with anorexia nervosa during the 3-year follow-up and in healthy controls.

Time (months)	N-Mid osteocalcin (ng/mL), mean ± SD	Median	CTX (pg/mL), mean ± SD	Median	N
0	22.33 ± 14.01	17.10	7792.54 ± 5427.72	6540.50	32
6	29.71 ± 24.13	18.74	5402.93 ± 2756.10	3799.00	15
12	31.71 ± 35.42	22.22	6280.66 ± 4579.28	4846.00	19
18	38.49 ± 34.74	27.30	5170.60 ± 3807.90	5170.50	16
24	37.85 ± 35.99	26.27	5919.54 ± 4017.27	4968.00	14
30	39.72 ± 41.33	18.25	6130.05 ± 3863.23	4833.00	11
36	25.85 ± 17.82	20.56	3782.51 ± 2235.90	3654.70	24
**Healthy controls**	56.68 ± 62.67	33.30	8203.38 ± 5035.92	7263.00	37

**Figure 6:**
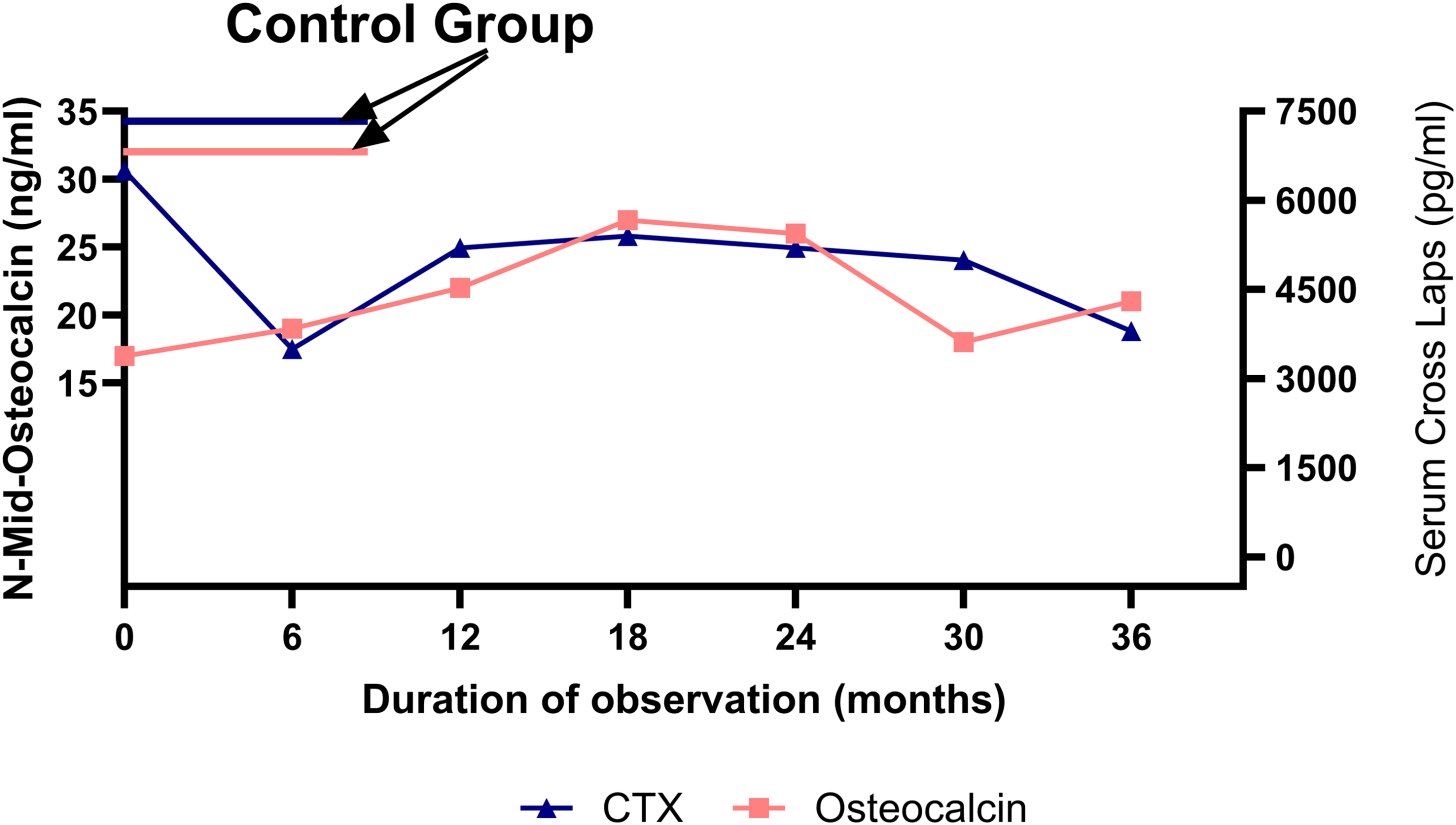
Baseline N-Mid osteocalcin and serum CrossLaps (CTX) levels in adolescents with anorexia nervosa compared with healthy controls, and their three-year longitudinal changes in the AN cohort.

BTMs changed over time in accordance with clinical improvement. Serum alkaline phosphatase and N-Mid Osteocalcin gradually increased, whereas PTH levels were variable and did not show consistent associations with other parameters. The duration of secondary amenorrhea was negatively associated with Z-score, suggesting a potential relationship between longer amenorrhea duration and lower bone mass. In contrast, improvements in BMI (used in correlation analyses) were not associated with Z-score recovery (**Table 6**, **Figure 6**).

## 4 Discussion

### 4.1 Bone density changes and hormonal interventions

In our cohort, the greatest decline in bone mineral density (BMD) among adolescent girls with anorexia nervosa (AN) was observed between 12 and 24 months of illness, with only partial recovery after 36 months of follow-up. This pattern was consistent across all subgroups defined by baseline Z-score (Ia, Ib, Ic), confirming that the early phase of the disorder represents a critical period of accelerated bone loss (15–18). The initial rapid decline in BMD is likely due to a combination of chronic undernutrition, hypogonadism, and hormonal disturbances affecting both bone formation and resorption (16).

Numerous reports in the literature describing densitometric assessments performed many years after AN onset emphasize that the main condition for BMD improvement in these patients is restoration of body weight and the resumption of menses; however, even in such cases, the improvement often remains only partial. Jagielska et al. performed densitometric studies in women 6–21 years after AN onset and demonstrated that despite clinical improvement, a significant proportion of patients still exhibited reduced BMD (19). Similarly, Herzog et al. reported persistent low bone density on average 11.7 years after the initial assessment (20). Achamrah et al., analyzing 160 patients over a three-year follow-up, showed that weight gain alone did not lead to full BMD normalization (21). Other studies also report only partial improvement in BMD over 2–3 years of follow-up (22–26). Long-term analyses covering up to 40 years indicated an approximately 46% increased risk of fractures in patients with AN compared to the general population (23).

Analysis of therapeutic subgroups in our cohort showed that girls receiving calcium and vitamin D_3_ supplementation combined with estrogen–progesterone therapy (group IIb) achieved greater improvement in Z-score after 36 months compared to pharmacologically untreated patients (group IIa). However, it should be emphasized that the groups differed at baseline regarding Z-score, age, and duration of secondary amenorrhea, and the allocation of treatment was non-randomized and based on clinical indications. Due to the small sample size and the lack of randomization, differences between IIa and IIb should be interpreted as descriptive observations rather than definitive evidence of treatment efficacy. In patients with the lowest BMD and the longest duration of secondary amenorrhea, an apparent improvement in bone mineral density was observed in those who received hormonal therapy; however, this observation should be interpreted cautiously due to confounding by indication and non-randomized treatment allocation. Although some natural fluctuations may occur within each cohort, the observed changes were clinically relevant and are consistent with the potential role of pharmacological treatment in selected high-risk patients. The results pertain to a highly selected group of patients, which may reduce the influence of psychological and behavioral factors on BMD but may also limit the generalizability to the broader population of girls with AN.

These findings are broadly consistent with previous studies demonstrating that physiological estrogen replacement can beneficially affect bone mineral density and support bone recovery in adolescents with AN, particularly in high-risk populations (17, 27, 28). However, while more recent data (2020–2025) partially support the benefit of estrogen therapy and supplementation, there is a lack of large, randomized clinical trials employing exactly the same combination and long-term follow-up (36 months) in adolescent AN patients. Hormonal therapy (including estrogen–progesterone regimens) was associated with improvement in BMD; however, this observation should be interpreted cautiously due to non-randomized treatment allocation and limited treatment duration (27, 29).

Literature data indicate that even with BMI normalization and nutritional therapy, the absence of restored gonadal function results in persistent BMD deficits (14, 17, 27). In summary, full restoration of bone mass requires recovery of gonadal function and adequate nutritional status, while in cases of persistent hypogonadism, estrogen replacement therapy may be necessary. Weight gain alone is insufficient.

In our cohort, increases in BMI were associated with recovery of hormonal parameters, including IGF-1 and estradiol, but were not directly correlated with improvements in BMD Z-scores. These findings suggest that skeletal recovery in adolescents with anorexia nervosa depends not only on weight restoration, but also on normalization of the endocrine milieu, particularly gonadal function. This is particularly evident during the early phase of weight restoration, when improvements in BMI are accompanied by recovery of hormonal parameters but not yet by measurable improvements in bone mineral density.

Our results highlight the potential significance of calcium metabolism parameters in evaluating bone changes in adolescents with AN. In our cohort, baseline serum calcium negatively correlated with Z-score, which may reflect increased bone resorption in patients with more severe undernutrition (15). Furthermore, a higher baseline urinary calcium-to-creatinine ratio was associated with both greater BMD loss and greater bone density improvement during the three-year follow-up. This apparent discrepancy may reflect dynamic bone turnover, where higher calcium levels indicate increased resorption at baseline but also identify patients with greater remodeling activity and potential for subsequent recovery. This suggests that even calcium values within the reference range may reflect active bone metabolism in adolescents with AN (15, 30). It should be emphasized that these observations are exploratory, based on a relatively small cohort, and should be interpreted with caution due to the non-randomized treatment allocation. Therefore, calcium parameters may potentially serve as additional indicators of bone turnover dynamics during the disease, but their prognostic significance requires confirmation in larger, prospective studies.

### 4.2 Hormonal regulation of bone metabolism

Hormones played a key role in maintaining skeletal health. Estradiol was positively correlated with BMI, FSH, and LH, and negatively with cortisol, whereas IGF-1 showed positive associations with BMI, LH, ALP, and osteocalcin, and negative associations with cortisol. These findings emphasize the interdependence of nutritional status, gonadal hormones, and the IGF-1 axis in regulating bone metabolism (25, 28).

Hypercortisolemia was associated with lower BMI, estradiol, IGF-1, FSH, and LH, consistent with its adaptive role in response to chronic energy deficiency, which contributes to metabolic homeostasis but has unfavorable effects on bone formation (31). The lack of significant correlations between estradiol, IGF-1, cortisol, and baseline Z-score suggests that bone deficits may appear before detectable hormonal disturbances or that illness duration plays a dominant role (31–33). Together, these findings support the concept that bone loss in AN results from the combined effects of nutritional deficiency, hypogonadism, and alterations in the IGF-1 axis, rather than from a single hormonal disturbance.

### 4.3 Bone turnover markers

BTMs provided additional insight. At baseline, osteocalcin levels were significantly lower in patients with AN compared to healthy controls (p = 0.002), whereas CTX did not differ significantly, indicating predominant impairment in bone formation with relatively preserved resorption (7, 13, 34–38). Over the three-year follow-up, osteocalcin progressively increased, peaking at 18–24 months, while CTX gradually declined, reflecting partial normalization of bone turnover. These trajectories support the model of low-turnover osteoporosis in AN, characterized by impaired bone formation as the dominant defect (39–42). The positive correlations between IGF-1 and formation markers, along with the negative correlations between cortisol, formation markers, and BMD, further underscore the importance of both IGF-1 deficiency and hypercortisolemia in the pathophysiology of bone loss in AN (43, 44).

In addition, baseline serum calcium and urinary calcium-to-creatinine ratio were significantly associated with BMD (Z-score) and 24-hour urinary cortisol levels. Higher levels of urinary cortisol were associated with increased urinary calcium excretion, which may indirectly reflect enhanced bone resorption, highlighting the effects of stress-axis activation on bone mineral homeostasis. Interestingly, we observed hypercalciuria in some patients despite serum cortisol levels remaining within the normal range, which may reflect increased activity of the stress axis and contribute to increased calcium loss and bone resorption in adolescents with AN.

### 4.4 Duration of secondary amenorrhea

The duration of secondary amenorrhea emerged as an important clinical correlate of lower BMDZ-scores in adolescent girls with anorexia nervosa. This finding underscores the pivotal role ofprolonged hypogonadism in the development of reduced BMD, consistent with prior research (27, 28, 32, 35, 36, 38, 41).

### 4.5 What the study adds

This study provides a three-year longitudinal observational dataset describing changes in bone mineral density, biochemical and hormonal parameters, and clinical characteristics in adolescent girls with anorexia nervosa.

Importantly, our findings suggest that weight restoration alone may not be sufficient for skeletal recovery. Although BMI improvement was associated with recovery of hormonal parameters, it was not directly associated with changes in BMD, indicating that the effect of nutritional rehabilitation on bone is likely mediated through endocrine normalization.

Furthermore, our results highlight that skeletal recovery may occur with a delay relative to metabolic and hormonal improvements, particularly during the early phase of weight restoration.

In addition, calcium homeostasis—both serum and urinary calcium—may reflect bone resorption and could be associated with skeletal recovery in adolescents with anorexia nervosa. The observed hypercalciuria, even in the presence of normal serum cortisol levels, may reflect a state of relative hypercortisolemia contributing to bone loss.

## 5 Conclusions

1. The most pronounced BMD loss in adolescent girls with AN occurs within the first 12–24 months of illness, with partial recovery observed after 36 months of follow-up.
2. Baseline serum calcium and urinary calcium-to-creatinine ratio may reflect bone turnover dynamics and could be associated with changes in BMD.
3. Endocrine regulators play a pivotal role in skeletal homeostasis: estradiol and IGF-1 enhance osteoblast differentiation and bone formation, whereas hypercortisolemia—absolute or relative—suppresses osteoblastic activity and promotes resorption.
4. Hormonal therapy (including estrogen–progesterone regimens) was associated with improvement in BMD; however, this observation should be interpreted cautiously due to non-randomized treatment allocation and limited treatment duration.
5. Bone turnover markers indicate that impaired bone formation predominates in AN; significant recovery appears to require simultaneous improvement in nutritional, hormonal, and metabolic status.
6. Longer duration of secondary amenorrhea was associated with lower BMD Z-scores in adolescent girls with anorexia nervosa.
7. Early densitometric assessment, monitoring of bone turnover markers, and integrated management (nutrition and hormonal recovery) may support BMD restoration.
8. Improvement in BMI was associated with recovery of hormonal parameters but not directly with BMD, suggesting that skeletal recovery is mediated by endocrine normalization and occurs with a delay relative to metabolic and hormonal changes during treatment, particularly during the early phase of weight restoration.

## 6 Limitations

The relatively small sample size and limited subgroup numbers reduce the statistical power of the study and require cautious interpretation of the results. Nevertheless, our observations are consistent with findings from previous studies conducted in similar cohorts, which support their clinical relevance. Although long-term studies in adolescents with anorexia nervosa are available, relatively few provide comprehensive longitudinal datasets integrating densitometric, hormonal, biochemical, and bone turnover parameters.

The exploratory correlations should therefore be interpreted as hypothesis-generating rather than confirmatory and require validation in larger prospective studies.

The retrospective design of the study limited the availability of complete data at all time points, particularly with respect to BMI-for-age Z-scores (zBMI), which could not be consistently calculated for all measurements. Therefore, correlation analyses were performed using absolute BMI values.

The lack of direct measurements of serum vitamin D concentrations limits the ability to fully evaluate its role. However, the supplementation used was modest and therefore unlikely to have been the sole factor responsible for the observed improvement in BMD.

The findings related to serum and urinary calcium parameters are promising but require further investigation in larger cohorts to confirm their potential prognostic value.

In summary, our observations suggest that integrated management including nutritional rehabilitation, calcium and vitamin D supplementation, and estrogen–progesterone therapy may support bone recovery in adolescent girls with anorexia nervosa. However, improvement in BMD appears to depend primarily on restoration of hormonal function, including normalization of IGF-1 levels and menstrual function, with BMI restoration acting as an indirect mediator.

## Data Availability

The original contributions presented in the study are included in the article/supplementary material. Further inquiries can be directed to the corresponding author.
